# Efficacy of glucosamine plus diacerein versus monotherapy of glucosamine: a double-blind, parallel randomized clinical trial

**DOI:** 10.1186/s13075-016-1124-9

**Published:** 2016-10-12

**Authors:** Jatupon Kongtharvonskul, Patarawan Woratanarat, Mark McEvoy, John Attia, Siwadol Wongsak, Viroj Kawinwonggowit, Ammarin Thakkinstian

**Affiliations:** 1 Section for Clinical Epidemiology and Biostatistics, Faculty of Medicine, Ramathibodi Hospital, Mahidol University, Bangkok, Thailand; 2 Department of Orthopedics, Faculty of Medicine, Ramathibodi Hospital, Mahidol University, Bangkok, Thailand; 3 Centre for Clinical Epidemiology and Biostatistics, University of Newcastle, Newcastle, NSW Australia; 4 Centre for Clinical Epidemiology and Biostatistics, School of Medicine and Public Health, University of Newcastle, Newcastle, NSW Australia; 5 Hunter Medical Research Institute, Newcastle, NSW Australia

**Keywords:** Glucosamine, Diacerein, Combined therapy, Monotherapy, Osteoarthritis, Knee

## Abstract

**Background:**

Patented crystalline glucosamine sulfate (pCGS) and diacerein monotherapy have been recommended for treatment of mild to moderate osteoarthritis (OA), but evidence of efficacy for combined treatments is lacking. Therefore, the aim of this study was to compare clinical outcomes (i.e., pain and Western Ontario and McMaster Universities Osteoarthritis Index [WOMAC] score) at 6 months as well as the safety profile of treatment with combined pCGS and diacerein versus pCGS alone.

**Methods:**

A double-blind, parallel randomized controlled superiority trial was conducted between August 2013 and August 2014 at Ramathibodi Hospital, Bangkok, Thailand. A total of 148 patients (74 patients in each group) was randomly allocated to receive pCGS plus diacerein or pCGS plus placebo daily. Adult patients with OA were eligible if they had a Kellgren-Lawrence grade of 2–3. The primary outcomes were visual analogue scale score (VAS) for pain and WOMAC subscores measured at 24 weeks after receiving treatment, using the intention-to-treat principle (nonresponder imputation).

**Results:**

Among the 148 patients in the study, mean age and body mass index were 60 years and 28.1 kg/m^2^, respectively. Mean VAS and minimal joint space width at baseline were 5.1 and 2.5 mm, respectively. The mean VAS values measured at 24 weeks were 2.97 and 2.88 in the pCGS plus diacerein and pCGS plus placebo groups, respectively. The estimated mean difference was 0.09 (95 % CI −0.75 to 0.94), which was not statistically significant (*P* = 0.710). In addition, the mean WOMAC total, pain, function, and stiffness scores for both groups were not significantly different, with corresponding means of 48.59, 12.02, 32.74, and 3.85 for the pCGS plus diacerein group and 48.69, 11.76, 32.47, and 4.16 for the pCGS plus placebo group. The risk of diarrhea and dyspepsia was very similar between the two groups, with risk ratios of 1.03 (95 % CI 0.56–1.89) and 0.91 (95 % CI 0.43–1.92), respectively.

**Conclusions:**

This study did not demonstrate that coadministration of diacerein with pCGS improves pain and WOMAC score compared with pCGS monotherapy in patients with mild to moderate OA of the knee.

**Trial registration:**

ClinicalTrials.gov identifier: NCT01906801. Registered on 20 July 2013.

**Electronic supplementary material:**

The online version of this article (doi:10.1186/s13075-016-1124-9) contains supplementary material, which is available to authorized users.

## Background

Osteoarthritis (OA), a degenerative joint disease, is the most common health problem in the United States [1]. According to the Global Burden of Disease 2013 project, musculoskeletal disorder contributes 6.8 % of the total disability-adjusted life-years(DALYs), with 10 % of this due to OA [2]. Increased longevity and obesity in most developed countries are expected to dramatically increase the incidence and prevalence of OA of the knee within the next decade [1, 3]. Current estimates indicate that the prevalence rates of knee OA are approximately 15 % in the United States [4] and about 34.5–45.6 % in elderly Thai [5]. It has been estimated that 40 % of the population aged over 65 years is affected by knee or hip symptomatic OA [6, 7].

Currently, there is no known cure for OA, and no intervention has been unequivocally demonstrated to delay disease progression before joint replacement surgery [8]. As for pharmacologic therapy, first-line drugs for OA are used purely for managing pain. Analgesic agents and nonsteroidal anti-inflammatory drugs (NSAIDs), including cyclooxygenase 2 inhibitors, are the most widely prescribed. However, the side effects of these treatments, which include an increased risk of cardiovascular events (e.g., heart attacks and stroke [9]), suggest that these drugs should be used with caution and should be avoided in patients with OA who have underlying cardiovascular disease [10]. Therefore, there remains a need for a therapeutic agent for OA that has symptom-modifying effects, a better safety profile, and positive (or at least no negative) effects on cartilage [11].

Patented crystalline glucosamine sulfate (pCGS) and diacerein are commonly used for treatment of symptomatic mild to moderate knee OA to relieve joint pain and delay joint destruction and cartilage loss. pCGS was developed as a prescription drug for OA in Europe and Asia, but it is available as an over-the-counter product in the United States and Australia. Diacerein is also available as an over-the-counter product in some countries in Asia, but not in other countries (e.g., Thailand) [8]. pCGS is found naturally in the human body, acting as one of the building blocks of cartilage and a precursor for glycosaminoglycan, a major component of joint cartilage [12]. Diacerein works by inhibiting interleukin-1, one of the first cytokines that induces fever, controls lymphocytes, increases the number of bone marrow cells, and causes degeneration of the bone joint [13].

The efficacy of pCGS and diacerein compared with placebo and active drugs has been estimated in systematic reviews and a network meta-analysis of randomized controlled trials (RCTs) [14–17]. The results of these investigations suggest that diacerein may be better than glucosamine for reducing pain, but both have similar efficacy for improving joint function. Because pCGS and diacerein have anabolic [18, 19] and catabolic effects [13], respectively, combining them may have synergetic effects and thus improve pain and function better than monotherapy. However, no RCT has directly compared the clinical outcomes between monotherapy (i.e., diacerein or pCGS) and a combination of the two drugs. We thus wanted to determine whether combined diacerein and pCGS is superior to pCGS alone. Therefore, we conducted a RCT with the aim of comparing clinical outcomes (i.e., pain and OA score) at 6 months as well as the safety profiles of treatment with combined pCGS and diacerein versus pCGS alone.

## Methods

### Trial design

The study design was a double-blind, parallel, randomized, controlled superiority trial. The trial was conducted at the orthopedics outpatient clinic of Ramathibodi Hospital, Bangkok, Thailand, between August 2013 and August 2014. It was conducted according to the original protocol regarding trial design, treatments, and outcome assessments, except dealing with missing data using imputation. The trial was also conducted and reported in accordance with the Consolidated Standards of Reporting Trials (CONSORT) statement.

### Eligibility criteria

Patients were recruited from the orthopedic outpatient clinic at Ramathibodi Hospital by orthopedic residents and staff between August 2013 and August 2014. Residents and staff were trained in how to recruit and inform patients about the trial. Patients were eligible if they met all of the following criteria:

- Diagnosed as having primary or secondary knee OA based on the clinical criteria of the American College of Rheumatology [20] (i.e., knee pain measured with a visual analogue scale [VAS] plus three of the following: aged 50 years or older, bony tenderness, stiffness lasting less than 30 minutes, bony enlargement, crepitus, or warm to touch)
- Had not received pCGS or diacerein within previous 6 months
- Had radiographic evidence of OA with a Kellgren-Lawrence grade of 2 or 3
- Were willing to participate and provided consent

Patients were excluded if they had any of the following:

- Had undergone knee replacement surgery
- Inflammatory arthritis (e.g., systemic lupus erythematosus, rheumatoid arthritis, gout) and posttraumatic arthritis
- Previous intra-articular treatment of the knee joint with any product (corticosteroids in the previous 2 months or hyaluronic acid in the previous 6 months)
- Gastrointestinal conditions (gastroesophageal reflux disorder, inflammatory bowel syndrome, peptic ulcer, and duodenal ulcer), renal disease, liver disease, or diabetes mellitus

### Treatment regimen and randomization

Eligible patients were randomly assigned to receive either a sachet of pCGS 1500 mg (Rottapharm Madaus, Monza, Italy) plus placebo once daily or pCGS 1500 mg plus diacerein 50 mg once daily (TRB Chemedica International S.A., Geneva, Switzerland) for 6 months. TRB Chemedica International prepared placebo capsules identically to diacerein by appearance, smell, and taste. Patients, physicians, assessors, and research nurses did not know which one was the active drug or the placebo.

A block randomization with a ratio of 1:1 was applied to generate a randomization list, with varying block sizes of 4–8. This procedure was prepared by a biostatistician (AT) who was not involved in patient recruitment or data collection. STATA version 13.0 software (StataCorp, College Station, TX, USA) was used to generate the random sequence lists [21], which were then prepared using coded drug packages and administered by a research nurse if patients met the inclusion criteria and had given informed consent. Patients might be prescribed other pain relief (acetaminophen 500 mg or NSAIDs), depending on the physician’s judgment. The use of NSAIDs could be started with ibuprofen 400 mg one tablet three times per day or naproxen 250 mg one tablet two times per day if patients were allergic to ibuprofen. The patients were provided with a diary to record their daily pain medication intake.

### Outcome measures

The primary outcomes of interest were pain score measured at 24 weeks using a VAS (ranging from 0 to 10, where higher score indicates greater pain) and the OA score measured at 24 weeks using the Thai version of the Western Ontario and McMaster Osteoarthritis Index (WOMAC) [22]. The WOMAC consists of 3 domains and 22 items comprising pain (5 items), function (15 items), and stiffness (2 items). Each item was graded from 0 to 10, with higher scores indicating more severe symptoms. Total and subdomain scores were calculated by summation of scores for relevant items. The total scores range from 0 to 220, where higher scores indicate more severe OA.

The secondary outcomes of interests were WOMAC subscores for pain, function, stiffness, and joint space width (JSW). The WOMAC scores were measured by a well-trained research assistant using WOMAC questionnaires at baseline and weeks 4, 8, 12, 16, 20, and 24 after treatment. JSW was assessed using weight-bearing metatarsophalangeal radiography at baseline, 12 weeks, and 24 weeks, and determined by computer-generated measurements taken from digitized images. JSW was defined as the distance from the distal femoral condyle to the proximal tibia, and it was measured by one orthopedist (JK) at 12 and 24 weeks after treatment. The intra- and interobserver reproducibility of this technique were considered to be acceptable, with an interobserver intraclass coefficient of correlation of 0.912 (0.887–0.931) and an intraobserver intraclass coefficient of correlation of 0.996 (0.991–0.998) [23]. In addition, adverse events, including gastrointestinal effects (i.e., dyspepsia and diarrhea), were assessed at each visit after treatment. All adverse events reported by the patients during the study treatment were recorded on their Case Report Form and classified in terms of type, time of onset, severity (mild, moderate, or severe), duration, and outcome. The physician asked the patient, “How well did you tolerate the test medication?” and recorded the patient’s response. All the information concerning expected adverse events was provided on the informed consent form. Information regarding other covariables, including age, sex, knee symptoms (i.e., warmth and stiffness), underlying disease (i.e., diabetes, hypertension, malnutrition, cardiovascular disease, and obesity as defined by body mass index [BMI] ≥30 mg/m^2^), and disease severity at baseline, was also collected.

### Statistical analysis

The sample size was calculated on the basis of a superiority trial detecting a mean difference in VAS between pCGS plus diacerein and pCGS plus placebo. For the meta-analysis [24], the mean and SD of VAS scores in the pCGS group were 4.78 and 1.9, respectively. Type I error with a two-sided test, power of test, and ratio of the treatment groups were set at 0.05, 0.80, and 1:1, respectively. The estimated sample size needed was 59 for each group to detect a mean difference in VAS score of 1 unit. Loss to follow-up was estimated at 20 %, which yielded a required sample size of 148 patients.

Data were described using frequency for categorical data and mean (SD) or median (range) where appropriate for continuous data. The distributions of these baseline characteristics were then explored. If the distributions were different between the two intervention groups (i.e., ≥10 % for binary/categorical variables and ≥1 of the pooled SD for continuous variables), these variables were then considered for adjustment in the main analysis.

Continuous data for both primary and secondary outcomes, including VAS pain score and WOMAC total scores and subscores at 24 weeks, were compared between treatment groups using a mixed linear regression analysis with a hierarchical approach, in which a subject variation term was fitted in the model as a random effect and treatment was considered as a fixed effect. Marginal treatment effects between treatments and times were then estimated and compared. Covariables at baseline were included if they were unequally distributed between two groups as mentioned above. The normality of residuals of the mixed model was then checked using normality plots (i.e., quantile of normal distribution) and the Shapiro-Wilk test. Diagnostic measures were explored if the assumption of normality was violated. The continuous outcomes were then transformed where appropriate to meet the assumption.

Secondary outcomes with dichotomous data (i.e., adverse events at 24 weeks) were compared between treatment groups using a mixed-effects Poisson regression analysis in which a subject variation was fitted in the model as a random effect and the treatment was considered as a fixed effect. The incidence of adverse events in both groups and a ratio of the incidence (i.e., a risk ratio [RR]) between treatments and times were then estimated and compared. Unbalanced covariables at baseline were also included in a Poisson regression model.

An intention-to-treat (ITT) approach was applied for all analyses if there was any evidence of a protocol violation. Missing outcome data were imputed for maintaining ITT using a multivariate normal regression analysis with 20 replications [25]. Complete data (i.e., age, sex, BMI, VAS, and WOMAC subdomain scores at baseline) were used to simultaneously predict VAS and WOMAC subdomain scores after treatments. All analyses were performed using STATA version 14.0 software [21]. The Bonferroni correction was applied to adjust for inflation of type I error from six outcomes and thus six multiple tests [26]. If a significance level for the whole family of tests was 0.05, then the Bonferroni-corrected threshold for individual tests was 0.0083.

## Results

### Patient characteristics

**Fig. 1:**
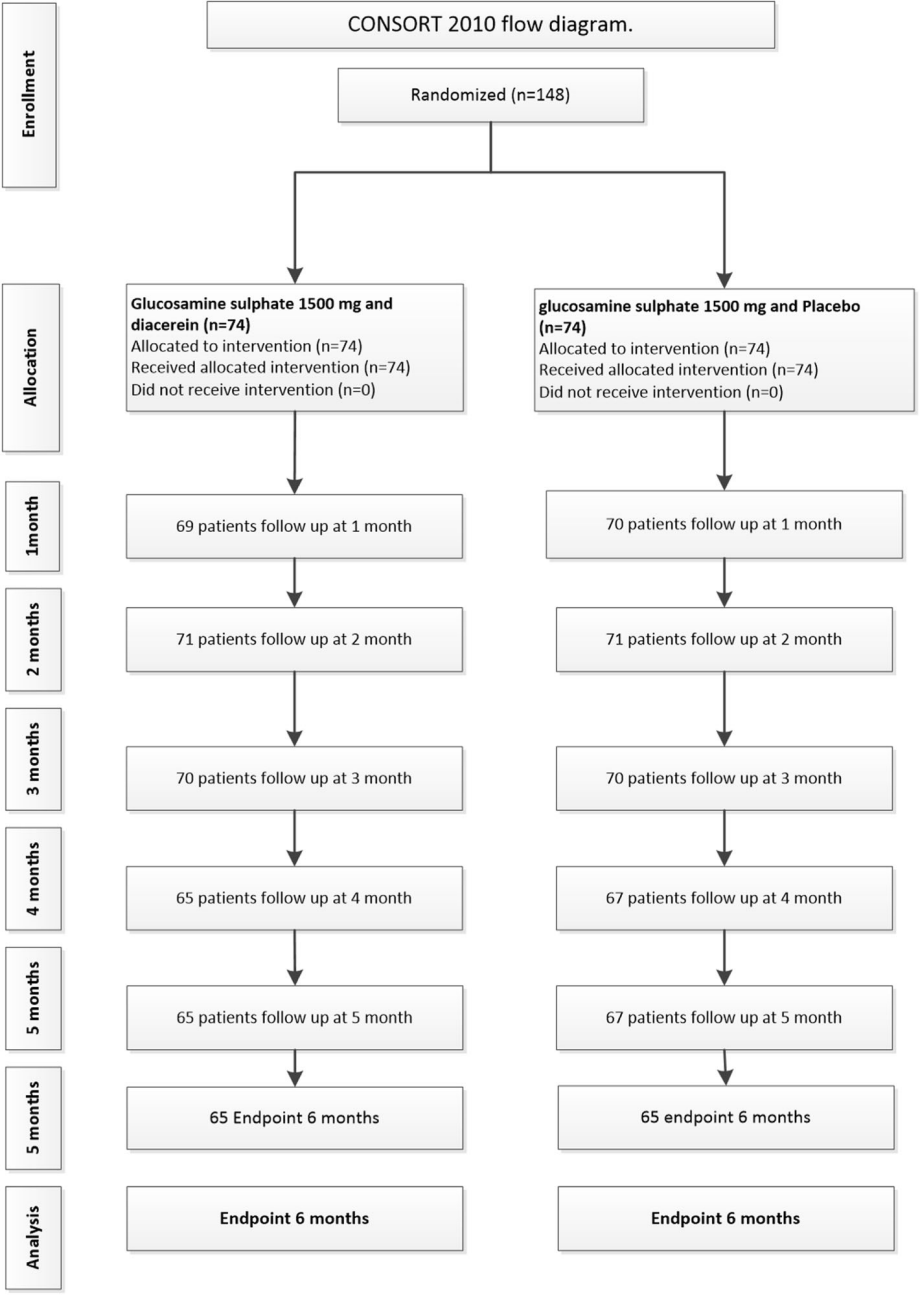
Consolidated Standards of Reporting Trials (CONSORT) 2010 flow diagram

A total of 148 patients was recruited and randomly allocated to treatment groups (see Fig. 1). Of these, 1 patient was ineligible because his pain scores on the VAS and WOMAC at baseline were 0, leaving 147 patients for assessment of clinical outcomes. Baseline characteristics were described, and their distributions between treatment groups were explored (see Table 1). In the pCGS plus diacerein group, the majority were female (83.1 %), the mean (±SD) age was 58.8 (±6.6) years, and the mean BMI was 28.9 (±5.3) kg/m^2^. The mean age in the pCGS plus placebo group was about 2.4 years older, and the mean BMI was about 1.7 kg/m^2^ lower, than in the pCGS plus diacerin group. The rest of the variables and disease severity at baseline, including pain, function, and stiffness scores were comparable between treatment groups. Thirteen patients in the pCGS plus placebo group had diabetes, as did eight patients in the pCGS plus diacerein group; there were no differences between treatment groups. The number of remaining pills and painkillers used, including NSAIDs and paracetamol, were recorded at every visit; there were no differences in rescue pain medication between treatment groups.

**Table 1:** Baseline characteristics of patients between treatment groups

Characteristics	Glucosamine sulfate plus diacerein (*n* = 74)	Glucosamine sulfate plus placebo (*n* = 74)
Age, years, mean (SD)	58.8 (6.6)	61.2 (7.3)
Sex, %		
Male	11 (14.9)	14 (18.9)
Female	63 (85.1)	60 (81.1)
BMI, kg/m^2^, mean (SD)	28.9 (5.3)	27.2 (4.2)
Knee symptoms, *n* (%)		
Warmth		
Yes	22 (30.14)	24 (32.43)
No	51 (69.86)	50 (67.57)
Stiffness		
Yes	39 (52.7)	33 (44.6)
No	35 (47.3)	41 (55.4)
Duration of symptoms before enrollment, months, median (range)	12 (2–120)	12 (2–120)
Drug allergy, *n* (%)		
Yes	15 (20.3)	15 (20.3)
No	59 (79.7)	59 (79.7)
Underlying disease, *n* (%)		
Diabetes		
Yes	8 (10.8)	13 (17.6)
No	65 (89.2)	61 (82.4)
Hypertension		
Yes	40 (54.1)	34 (45.9)
No	34 (45.9)	40 (54.1)
Dyspepsia		
Yes	8 (11.76)	7 (9.86)
No	60 (88.24)	64 (90.14)
Other disease		
Yes	34 (45.9)	32 (43.2)
No	40 (54.1)	42 (56.8)
Defecation		
Normal	59 (79.7)	54 (73)
Abnormal	15 (20.3)	20 (27)
Smoking		
Yes	1 (1.4)	3 (4)
No	73 (98.6)	71 (96)
Alcohol drinking		
Yes	7 (9.5)	3 (4)
No	67 (90.5)	71 (96)
Family history of OA knee		
Yes	25 (33.8)	31 (41.9)
No	49 (66.2)	43 (58.1)
Quadriceps exercises		
Yes	46 (62.2)	48 (64.9)
No	28 (37.8)	26 (35.1)
Kellgren-Lawrence grade 2, %	81.1	76.4
VAS pain score, mean (SD)	5.01 (2.55)	5.05 (2.61)
WOMAC score		
Total, mean (SD)	82.3 (47.3)	81.4 (44.1)
Pain, mean (SD)	21.3 (11.8)	21.1 (12.3)
Stiffness, median (range)	6.5 (0–20)	4.5 (0–20)
Function, mean (SD)	54.2 (32.7)	54.4 (29.3)
Joint space width, mm, mean (SD)		
Medial minimal width, right	2.98 (0.82)	2.82 (0.84)
Lateral minimal width, right	4.25 (1.28)	4.32 (1.12)
Medial minimal width, left	2.83 (0.84)	2.90 (0.78)
Lateral minimal width, left	4.35 (1.10)	4.28 (1.15)

*Abbreviations: BMI* Body mass index, *OA* Osteoarthritis, *VAS* Visual analogue scale, *WOMAC* Western Ontario and McMaster Universities Osteoarthritis Index

Patient compliance with the allocated treatments, measured by counting the number of capsules at each visit, ranged from 88 % to 100 % in the pCGS plus diacerein group and from 79 % to 100 % in the pCGS plus placebo group. A total of 18 participants (9 per group) did not finish the study at 6 months. When we compared this group with the 130 participants (65 per group) who finished the study at 6 months, we observed no differences in baseline characteristics (see Additional file 1: Table S1). Loss to follow-up in these two corresponding groups ranged from 1.4 % to 12.2 %. These outcome data of 46 patients were then imputed using complete baseline data with 20 replications. These imputed and complete outcome data of 147 patients were used for further analyses using the ITT approach.

### VAS pain score

Applying a mixed-effects regression model with adjustments for age and BMI yielded mean VAS scores at 24 weeks of 2.97 (95 % CI 2.38–3.56) and 2.88 (95 % CI 2.29–3.47) in the pCGS plus diacerein and glucosamine plus placebo groups, respectively (see Table 2). This indicated no significant difference between the two groups, with an estimated mean difference of 0.09 (95 % CI −0.75 to 0.94).

**Table 2:** VAS scores, WOMAC total scores, WOMAC subscores, and joint space width at 6-month follow-up

Outcome at last follow-up	Treatment		Mean differences between groups	95 % CI	*P* value
	Glucosamine plus diacerein	Glucosamine plus placebo			
VAS score	2.97 (2.38–3.56)	2.88 (2.29–3.47)	0.09	−0.75, 0.94	0.710
WOMAC scores					
Total	48.59 (38.30–58.89)	48.69 (38.34–59.05)	−0.10	−14.95, 14.75	0.990
Pain	12.02 (9.53–14.52)	11.76 (9.25–14.27)	0.26	−3.34, 3.86	0.887
Stiffness	3.85 (2.79–4.91)	4.16 (3.07–5.26)	−0.32	−1.87, 1.24	0.687
Function	32.74 (25.70–39.79)	32.74 (25.66–39.82)	0.01	−10.15, 10.16	0.999
JSW, mm	2.63 (2.41–2.84)	2.59 (2.37–2.81)	0.04	−0.35, 0.27	0.803

*Abbreviations: JSW* Joint space width, *VAS* Visual analogue scale, *WOMAC* Western Ontario and McMaster Universities Osteoarthritis Index

**Fig. 2:**
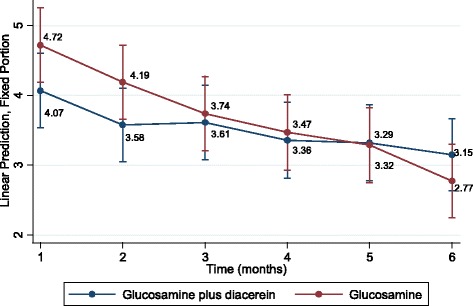
Mean visual analogue scale scores by treatment group and time

The mean VAS scores were plotted by treatment and time, which indicated declining VAS scores after treatment in both groups (see Fig. 2). The mean VAS scores in the pCGS plus diacerein group at 1, 2, 3, 4, 5, and 6 months were 4.07, 3.58, 3.61, 3.36, 3.32, and 3.15, respectively; the corresponding values in the pCGS and placebo group were 4.72, 4.19, 3.74, 3.47, 3.29, and 2.77.

Post hoc analysis was performed in a subgroup of patients whose VAS pain scores at baseline were 5 or higher. The overall mean VAS scores were 6.72 ± 1.69 and 6.92 ± 1.56 in the combined treatment and monotherapy groups, respectively, but there was no statistically significant difference (*P* = 0.607).

### WOMAC total score

Applying the mixed-effects regression model with adjustments for age and BMI yielded mean WOMAC total scores at 24 weeks of 48.59 (95 % CI 38.30–58.89) and 48.69 (95 % CI 38.34–59.05) in the pCGS plus diacerein and glucosamine plus placebo groups, respectively (see Table 2). This indicated no significant difference between the two groups, with an estimated mean difference of −0.1 (95 % CI −14.95 to 14.75).

**Fig. 3:**
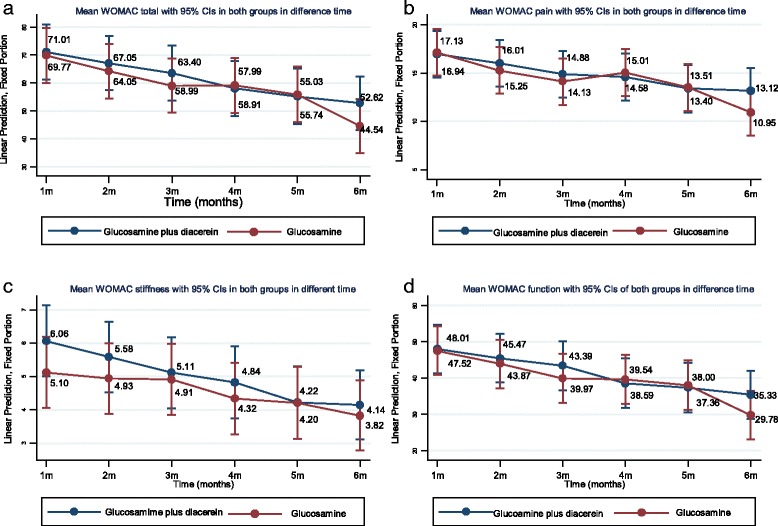
Western Ontario and McMaster Universities Osteoarthritis Index mean total scores and subscores by treatment group and time

Mean WOMAC total scores were plotted by treatment and time, which indicated declining WOMAC scores in both treatment groups (see Fig. 3a). The mixed-effects regression model indicated no significant difference between the two groups at each distinct time point.

### WOMAC pain score

Applying the mixed-effects regression model with adjustments for age and BMI yielded mean WOMAC pain scores at 24 weeks of 12.02 (95 % CI 9.53–14.52) and 11.76 (95 % CI 9.25–14.27) in the pCGS plus diacerein and glucosamine plus placebo groups, respectively (see Table 2). This indicated no significant difference between the two groups, with an estimated mean difference of 0.26 (95 % CI −3.34 to 3.86). Mean WOMAC pain scores were plotted by treatment and time, which indicated declining WOMAC scores in both treatment groups (see Fig. 3b).

### WOMAC stiffness score

The mixed-effects regression model with adjustments for age and BMI yielded mean WOMAC stiffness scores at 24 weeks of 3.85 (95 % CI 2.79–4.91) and 4.16 (95 % CI 3.07–5.26) in the pCGS plus diacerein and glucosamine plus placebo groups, respectively (see Table 2). This indicated no significant difference between the two groups, with an estimated mean difference of −0.32 (95 % CI −1.87 to 1.24). Mean WOMAC stiffness scores were plotted by treatment and time, which indicated declining WOMAC scores in both treatment groups (see Fig. 3c).

### WOMAC function score

The mixed-effects regression model with adjustments for age and BMI yielded mean WOMAC function scores at 24 weeks of 32.74 (95 % CI 25.70–39.79) and 32.74 (95 % CI 25.66–39.82) in the pCGS plus diacerein and glucosamine plus placebo groups, respectively (see Table 2). This indicated no significant difference between the two groups, with an estimated mean difference of 0.01 (95 % CI −10.15 to 10.16).

Mean WOMAC function scores decreased over time for both treatments (see Fig. 3d). Applying the mixed-effects regression model indicated no significant difference between the two groups at each distinct time point.

### Minimal joint space width

**Fig. 4:**
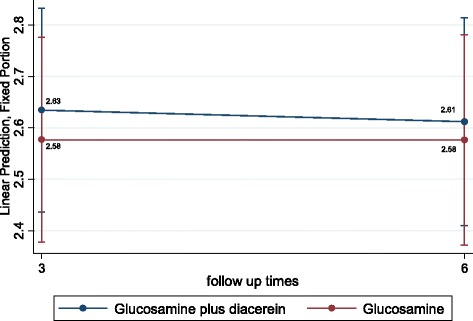
Mean difference of joint space width between glucosamine plus diacerein and glucosamine groups at different time points

Using an ITT analysis approach and applying the mixed-effects regression model with adjustments for age and BMI yielded mean JSWs at 24 weeks of 2.63 mm (95 % CI 2.41–2.84) and 2.59 mm (95 % CI 2.37–2.81) in the pCGS plus diacerein and glucosamine plus placebo groups, respectively (see Fig. 4). This indicated no significant difference between the two groups, with an estimated mean difference of 0.04 mm (95 % CI −0.35 to 0.27).

### Adverse events

The mixed-effects Poisson regression model was applied to estimate the risk of occurrence of dyspepsia and diarrhea with adjustments for age and BMI. The estimated risk of diarrhea after treatment was very close between the two groups (0.084 [0.051–0.136] for pCGS plus diacerein vs. 0.081 [0.048–0.138] for pCGS plus placebo) with an RR of 1.026 (95 % CI 0.559–1.885) (see Table 3). The risks of dyspepsia were also very similar (i.e., 0.030 [0.015, 0.059] vs 0.033 [0.017, 0.066]) with an RR of 0.910 (95 % CI 0.432–1.918).

**Table 3:** Incidence and risk ratio of adverse events between the two treatment groups

Outcomes at 6-month follow-up	Treatment		RR	95 % CI	*P* value
	Glucosamine plus diacerein	Glucosamine plus placebo			
Diarrhea	0.84 (0.05–0.14)	0.81 (0.05–0.14)	1.03	0.56–1.89	0.932
Dyspepsia	0.03 (0.02–0.06)	0.03 (0.02–0.07)	0.91	0.43–1.92	0.805

*RR* Risk ratio

The most common adverse event was abnormal urine (red or orange color), seen in 87.7 % of patients receiving pCGS plus diacerein and in 66.2 % of the patients receiving pCGS plus placebo. For approximately one-third of the patients, the adverse events were related to the gastrointestinal system (diarrhea, gastritis, constipation, and nausea); however, there were no significant differences between groups in this regard. Approximately 10 % of patients in both groups reported skin reactions. No adverse events led to the dropout of any patient or discontinuation of any medication. No deaths occurred in this study. Parameters determined on the basis of vital signs and physical examinations were similar in both groups. The consumption of rescue medication in this study (i.e., other pain medication) was low and similar between the two groups.

## Discussion

We conducted a double-blind RCT to compare the efficacy of the combination of pCGS plus diacerein with pCGS plus placebo in the treatment of knee OA. Our findings suggest that combined treatment does not reduce VAS pain score, WOMAC total score, or WOMAC subscores compared with pCGS monotherapy in patients with mild to moderate knee OA. Although the efficacy in both treatment groups did not differ, clinical signs and symptoms were improved in both treatment groups at 12–24 weeks, and this was particularly evident in the pCGS plus placebo group.

pCGS, which comprises essential components of the proteoglycans in normal cartilage, is found naturally in the knee joint of the human body. With its possible anabolic effect, it is used for inhibition of metalloproteinase activity, prostaglandin E_2_ release, nitric oxide production, degradation of glycosaminoglycans, and stimulation of the synthesis of hyaluronic acid in the joint [12, 27]. It has a slow onset of response, provides long-lasting pain relief and functional improvement, and delays progression of the joint space [28, 29] in OA of the knee [12, 30, 31]. Diacerein may be beneficial for OA in that it inhibits interleukin-1, controls lymphocytes, increases the number of bone marrow cells, and reduces degeneration of the bone joint [13]. As a result, diacerein is also claimed to improve pain and function in OA of the knee [30, 32–34]. In a recent animal study, researchers found chondroprotective effects of diacerein and pCGS, but a better range of motion of the knee joint was found in response to diacerein than to pCGS [35]. With the different mechanisms of action of pCGS and diacerein, it could be expected that combined treatments should result in synergetic effects.

Our findings are similar to those of a previous study in which researchers compared combined pCGS plus chondroitin sulfate with pCGS or chondroitin sulfate alone in patients with OA of the knee whose Kellgren-Lawrence grade was 2–3 [31]. That study was later combined in a network meta-analysis [36], which showed similar results. Although the potential synergistic effects derived from a pharmacologic study of pCGS and diacerein [27] looked promising, our findings indicate that combined treatments did not provide any benefit over monotherapy.

### Strengths and limitations

To the best of our knowledge, this is the first double-blind RCT designed to assess the effects of combined pCGS plus diacerein versus pCGS monotherapy with 6 months of follow-up. The active treatments, both capsules and sachets, were identical in appearance and were administered to patients using coded drug packs; therefore, patients, investigators, and outcome assessors were truly blinded. We considered the most relevant outcomes, including subjective (i.e., VAS pain score, WOMAC total score, and WOMAC subscores) and objective (i.e., minimal tibiofemoral JSW) measures. In addition, all possible adverse effects were collected. Drug compliance was reasonably high, ranging from 88 % to 100 % and 79 % to 100 % in the combined treatment and monotherapy groups, respectively (*P* = 0.133). Cointervention with additional pain medications was also similar at 17.6 % versus 21.6 % in the combined treatment and monotherapy groups, respectively (*P* = 0.534). We applied an ITT analysis by considering all patients in the groups to which they were originally randomly allocated, thus minimizing bias.

Our study has some limitations. The dosage of diacerein that we used was 50 mg in the combined treatment group because the side effect of diacerein has been shown to have a correlation to the drug dosage in prevention of drug withdrawal, according to a previous study in which researchers compared diacerein in different dosages (50, 100, and 150 mg) with placebo. The highest safety profile is at a dose of 50 mg/day, and we did not up-titrate the dose. However, the positive effects on VAS pain symptoms are decreased in a dose-dependent manner as the dose is decreased [34]. This could explain the lack of difference between the two treatment groups.

The sample size calculation was computed to assess primary outcomes between groups, but it may not be generalized to assessment of secondary outcomes; therefore, statistical insignificance might be due to the risk of type II errors. We considered mostly patients with knee OA with mild to moderate pain scores at baseline, which might have made it difficult to detect the benefits of combined treatment. In addition, our patients with knee OA were mainly diagnosed. The uncertain clinical diagnosis and classification may affect the outcomes of clinical studies [37–41]. There is a widespread belief that there is a high discordance between clinical and radiographic knee OA. In an attempt to overcome this problem, we included the participants in our study on the basis of American College of Rheumatology criteria [20] for diagnosis of knee OA with the radiographic criteria of Kellgren-Lawrence grade 2–3 [42]. However, the Kellgren-Lawrence classification strongly depends on adequate patient positioning when taking x-rays. It is also not specific to cartilage loss. Magnetic resonance imaging (MRI) provides somewhat superior sensitivity to change compared with commonly used radiographs, and it has recently provided non-location-dependent measures of cartilage thickness loss and gain, which are potentially more sensitive in detecting symptomatic slow-acting drug for osteoarthritis effects than radiographic JSW. The cost of MRI is about 20 times greater than that of radiographs; therefore, we used radiographically measured JSW with strict positioning to obtain the highest-quality outcome measurements. Moreover, knee pain and function of patients with these inclusion criteria, which we assessed using subjective outcome VAS and WOMAC scores, may be imprecise owing to the nonspecific nature of knee pain (e.g., non-OA pathology such as tendinitis or muscle strain, referred pain, and nonphysical pain such as depression), and all these factors can coexist at the same time, composing multiple layers of causality of knee pain [37].

The potential sources of bias that could substantially impact interpretation of the trial were noncompliance, cointervention, and contamination. For cointervention and contamination, there were no reports of use of nonprotocol medications or other cotreatments, which meant cointervention or contamination between the groups was unlikely. However, the data were analyzed by the ITT method. As for compliance, missing outcome data were imputed using a multivariate normal regression analysis with 20 replications. Complete data (i.e., age, sex, BMI, VAS and WOMAC subdomain scores at baseline) were used to simultaneously predict VAS and WOMAC subdomain scores after treatment.

We measured outcomes over 6 months owing to time restrictions, making the study a short-term assessment. However, according to previous studies of both drugs, there have been long-term effects lasting up to about 1 year (longest effect lasted 3 years). There were also sustained effects lasting longer than 3 months; therefore, when assessing stratified patients with knee OA in a subgroup with longer follow-up times, a sustained effect could be considered to deduce the benefits of combined treatment. Replication using larger samples with repeated measurements might show greater, more conclusive differences in all possible outcomes over time between the two groups. This information could be used to more properly address the treatment effects. Moreover, the results derived from this study were based on plain radiographs, which may not be sufficient to assess knee cartilage, the major component that responds to both drugs (based on the JSW of the knee joint). MRI may better facilitate the assessment of cartilage changes in the knee joint in the next study because MRI is much more sensitive than plain radiography. However, in this study, we assessed JSW at 3 and 6 months. These time periods may not be adequate for assessing changes in JSW and may be a reason for the insignificant results regarding radiographic changes in JSW. In postmarketing data, elevations in serum liver enzymes and acute hepatic injury have been recorded. In this study, although participants with preexisting liver problems were excluded, there were no measures to monitor liver toxicity; therefore, we were unable to detect any potential liver-related adverse drug events. Finally, there are various generic preparations of glucosamine that were approximately five times cheaper than the original ones. Bioequivalence trials between original and generic glucosamine samples should be conducted.

## Conclusions

This study did not demonstrate that coadministration of diacerein with pCGS improves pain and WOMAC scores compared with pCGS monotherapy in patients with mild to moderate OA with Kellgren-Lawrence grade 2–3. Both combined therapy and monotherapy can significantly reduce VAS pain and WOMAC function scores after 3–6 months compared with baseline.

## Additional file

Additional file 1: Table S1. Baseline characteristics of patients with complete and incomplete follow-up between treatment groups. (DOCX 16 kb)

## Abbreviations

BMI: Body mass index
CONSORT: Consolidated Standards of Reporting Trials
DALY: Disability-adjusted life-year
ITT: Intention to treat
JSW: Joint space width
MRI: Magnetic resonance imaging
NSAID: Nonsteroidal anti-inflammatory drug
OA: Osteoarthritis
pCGS: Patented crystalline glucosamine sulfate
RCT: Randomized controlled trial
RR: Risk ratio
VAS: Visual analogue scale
WOMAC: Western Ontario and McMaster Universities Osteoarthritis Index
