# Supplementary material for: Efficacy of glucosamine plus diacerein versus monotherapy of glucosamine: a double-blind, parallel randomized clinical trial
Source: Arthritis Res Ther. 2016 Oct 12;18:233. doi: 10.1186/s13075-016-1124-9 (PMC5059980; doi:10.1186/s13075-016-1124-9)
Supplement: Additional file 1: Table S1. — Baseline characteristics of patients with complete and incomplete follow-up between treatment groups. (DOCX 16 kb) [file 13075_2016_1124_MOESM1_ESM.docx]

Additional file 1: Table S1 Baseline characteristics of patients with complete and incomplete follow up between treatment groups

| Characteristics | Glucosamine sulfate plus diacerein  (n=74) | | Glucosamine sulfate plus placebo  (n=74) | |
| --- | --- | --- | --- | --- |
|  | Complete f/u (n=65) | Loss f/u (n=9) | Complete f/u (n=65) | Loss f/u (n=9) |
| Age (year), mean(SD) | 58.8 (6.6) | 59.3(7.2) | 62.1 (7.1) | 54.9 (5.8) |
| Sex (%) |  |  |  |  |
| Male | 11 (16.9) | 0 (0) | 12 (18.5) | 2 (22.2) |
| Female | 54 (83.1) | 9 (100) | 53 (81.5) | 7 (77.8) |
| BMI (kg/m^2^), mean(SD) | 28.8 (5.4) | 29.9 (5.3) | 27.0 (4.3) | 28.8 (3.3) |
| Pain VAS score, mean (SD) | 4.87 (2.54) | 6.06 (2.51) | 5.04 (2.68) | 5.08 (2.19) |
| WOMAC questionnaire, mean (SD) | 79.89 (47.65) | 100 (42.67) | 82.65 (43.56) | 72.67 (49.95) |
| pain WOMAC, mean (SD) | 20.85(11.84) | 24.89 (11.84) | 21 (12.04) | 22.11 (14.60) |
| Stiffness WOMAC , median (range) | 6.74 (6.11) | 7.56 (5.55) | 6.2 (5.82) | 4.11 (3.26) |
| Function WOMAC, mean (SD) | 52.31 (33.05) | 67.56 (28.31) | 55.45 (28.77) | 46.44 (33.97) |
| Medial minimal joint space width (right), mean (SD) | 2.95 (0.80) | 3.17 (0.94) | 2.80 (0.83) | 2.86 (1.02) |
| Lateral minimal joint space width (right), mean (SD) | 4.30 (1.31) | 3.94 (0.97) | 4.25 (0.95) | 4.81 (2.02) |
| Medial minimal joint space width (left), mean (SD) | 2.79 (0.79) | 3.11 (1.15) | 2.92 (0.77) | 2.79 (1.12) |
| Lateral minimal joint space width (left), mean (SD) | 4.34 (1.15) | 4.4 (0.67) | 4.23 (1.16) | 4.51 (1.25) |
